# Supplementary material for: Mapping protein states and interactions across the tree of life with co-fractionation mass spectrometry
Source: Nat Commun. 2023 Dec 15;14:8365. doi: 10.1038/s41467-023-44139-5 (PMC10724252; doi:10.1038/s41467-023-44139-5)
Supplement: Supplementary file 1 — Supplementary Information [file 41467_2023_44139_MOESM1_ESM.pdf]

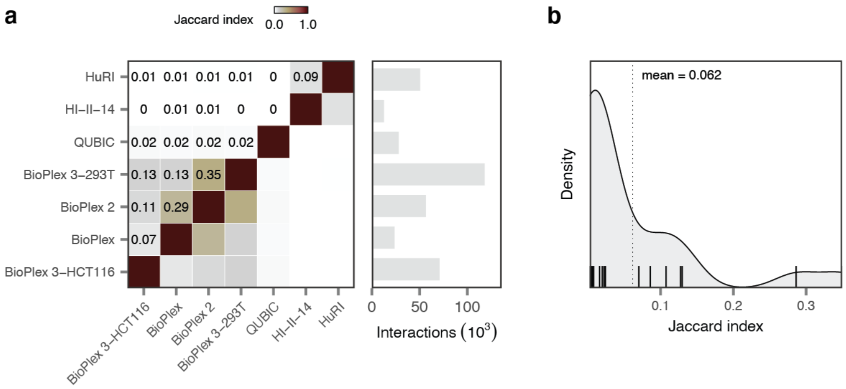

Supplementary Fig. 1 | Limited overlap between human interactome screens.

a, Overlap between high-throughput screens of the human interactome[1–6] performed using Y2H or AP-MS since 2014, as quantified by the Jaccard index. Right, total number of interactions recovered per screen.

b, Density plot showing the distribution of pairwise Jaccard indices between human interactome screens. Individual pairs of networks are shown as ticks along the x-axis. Vertical dotted line shows the mean Jaccard index across all network pairs.

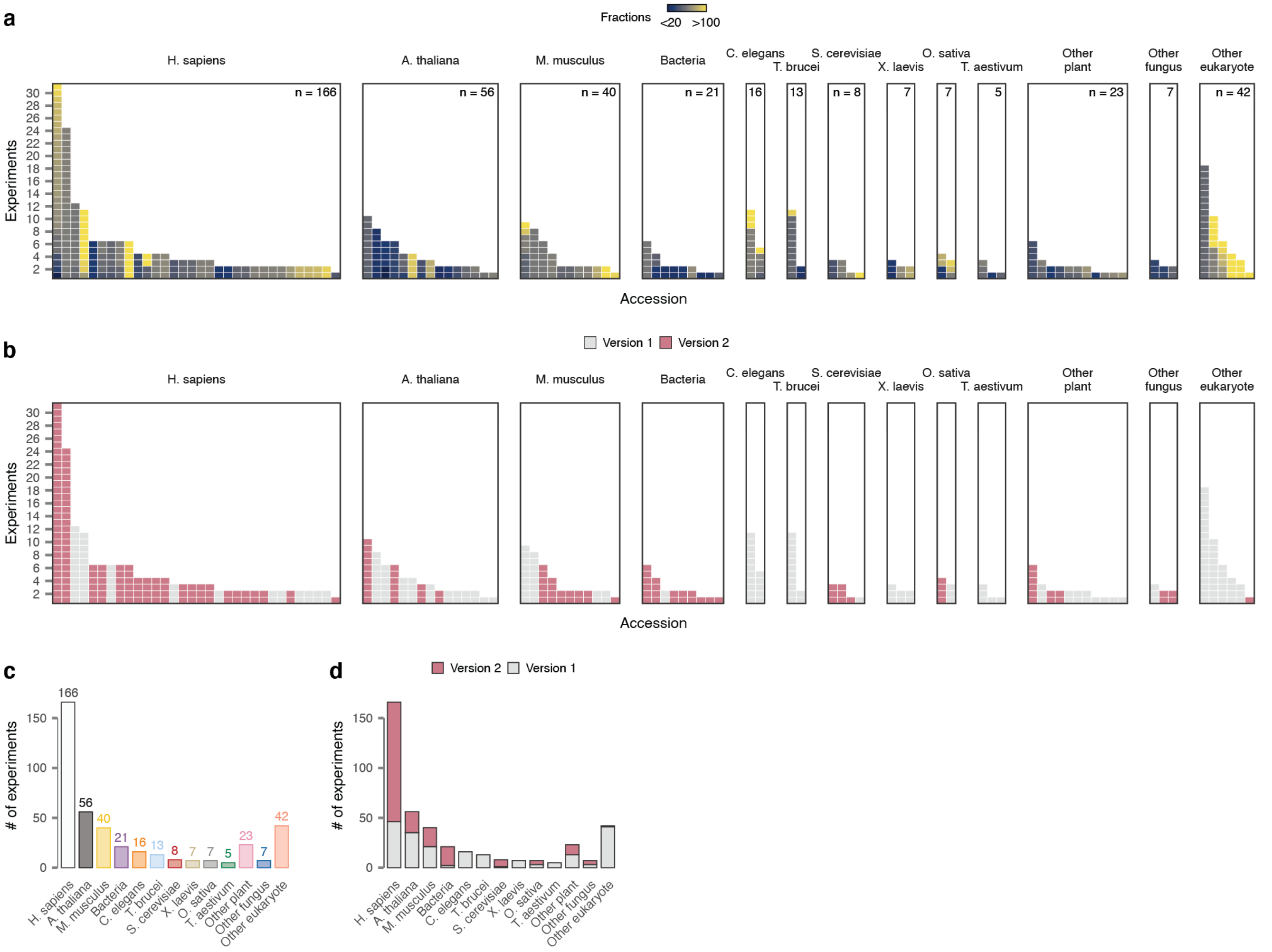

Supplementary Fig. 2 | Overview of CFdb.
a, Overview of the 411 CF-MS experiments in CFdb. Each column shows a single MassIVE or PRIDE accession, and cells represent CF-MS experiments. Cells are shaded by the number of fractions collected in each experiment.
b, As in **a,** but highlighting experiments that have been added since the original meta-analysis[7].
c, Number of experiments per species or major phylogenetic group.
d, As in **c,** but highlighting experiments that have been added since the original meta-analysis.

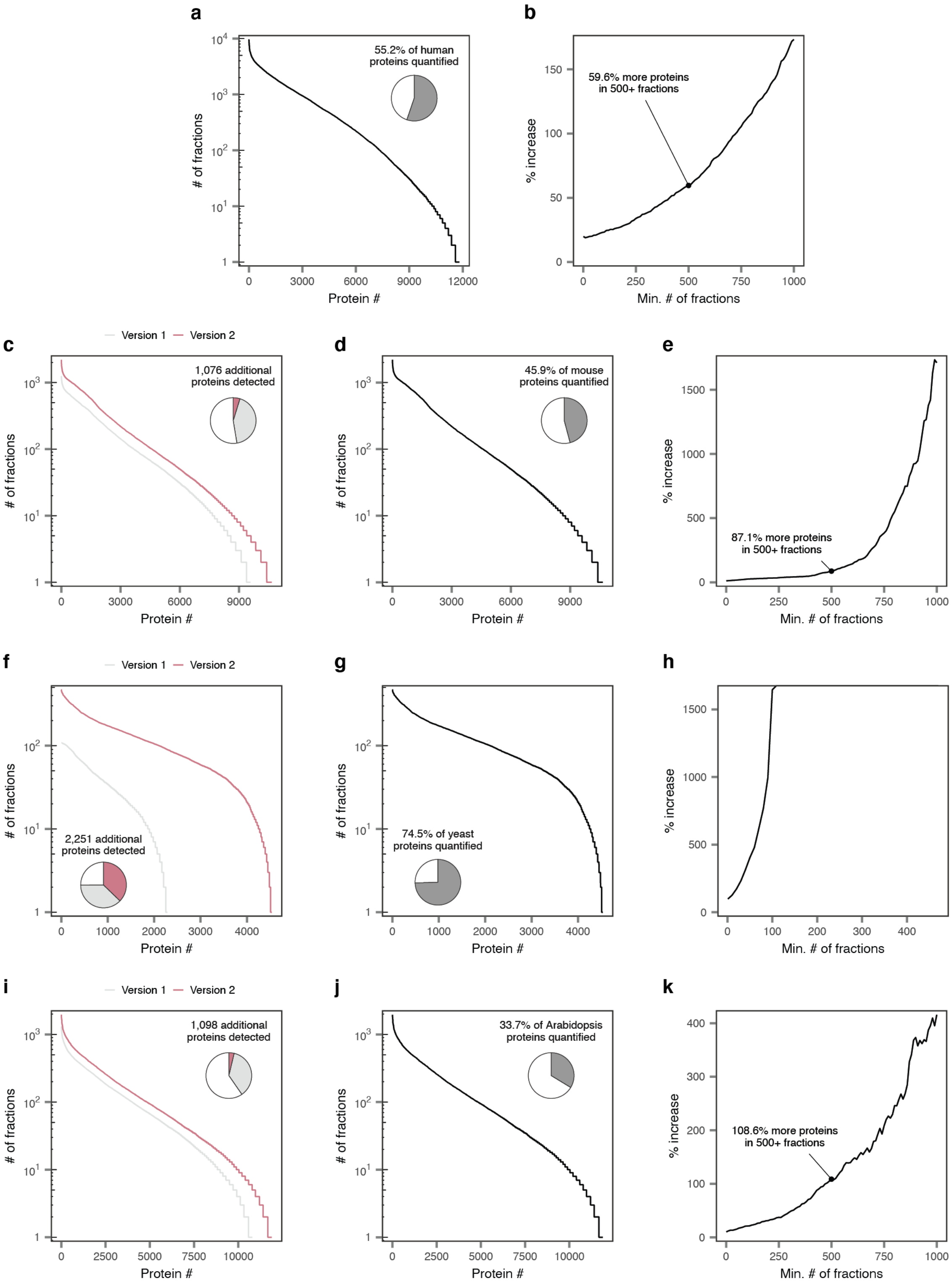
a
55.2% of human proteins quantified
# of fractions
Protein #
b
59.6% more proteins in 500+ fractions
% increase
Min. # of fractions
c
Version 1
Version 2
1,076 additional proteins detected
# of fractions
Protein #
d
45.9% of mouse proteins quantified
# of fractions
Protein #
e
87.1% more proteins in 500+ fractions
% increase
Min. # of fractions
f
Version 1
Version 2
2,251 additional proteins detected
# of fractions
Protein #
g
74.5% of yeast proteins quantified
# of fractions
Protein #
h
% increase
Min. # of fractions
i
Version 1
Version 2
1,098 additional proteins detected
# of fractions
Protein #
j
33.7% of Arabidopsis proteins quantified
# of fractions
Protein #
k
108.6% more proteins in 500+ fractions
% increase
Min. # of fractions

Supplementary Fig. 3 | Expanded resources of CF-MS data for human, mouse, yeast, and *Arabidopsis*.

a, Cumulative distribution function showing the number of fractions in which each human protein was quantified. Inset pie chart shows the total proportion of human proteins that were detected in at least one fraction.

b, Percentage increase in the number of human proteins quantified in at least *n* fractions ("well-quantified proteins"), for any value of *n*. The number of human proteins quantified in at least 500 fractions is highlighted.

c, Cumulative distribution function showing the number of fractions in which each mouse protein was quantified, in either the original meta-analysis (light grey) or CFdb (pink). Inset pie chart shows the total proportion of mouse proteins that were detected in at least one fraction, shown separately for the original meta-analysis versus CFdb.

d, As in **a,** but for mouse proteins.

e, As in **b,** but for mouse proteins.

f-h, As in **c-e,** but for yeast proteins.

i-k, As in **c-e,** but for *Arabidopsis* proteins.

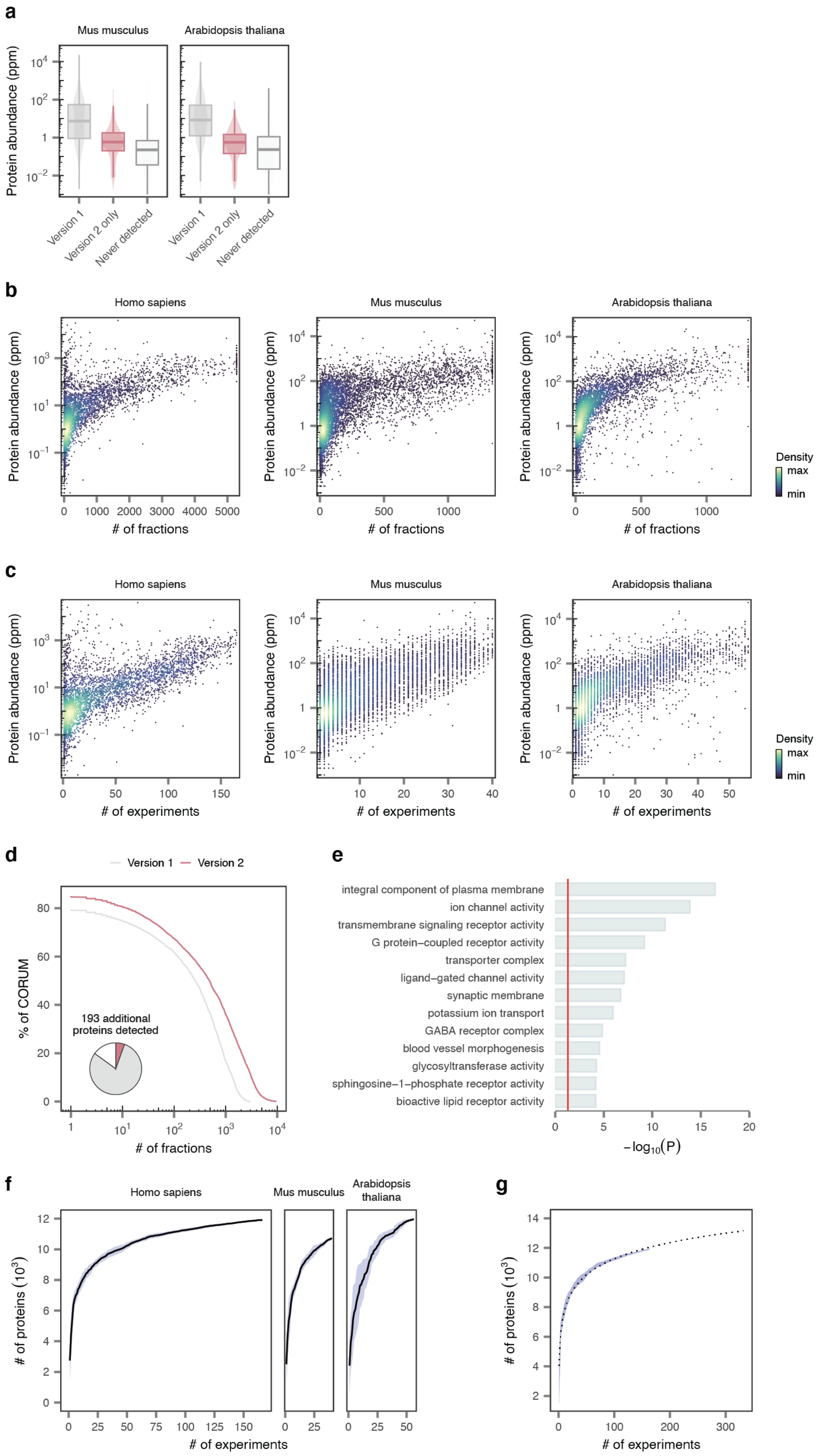
a
Mus musculus
Arabidopsis thaliana
Protein abundance (ppm)
Version 1
Version 2 only
Never detected
b
Homo sapiens
Mus musculus
Arabidopsis thaliana
# of fractions
Density
max
min
c
# of experiments
d
Version 1
Version 2
% of CORUM
193 additional proteins detected
e
integral component of plasma membrane
ion channel activity
transmembrane signaling receptor activity
G protein−coupled receptor activity
transporter complex
ligand−gated channel activity
synaptic membrane
potassium ion transport
GABA receptor complex
blood vessel morphogenesis
glycosyltransferase activity
sphingosine−1−phosphate receptor activity
bioactive lipid receptor activity
−log10(P)
f
# of proteins (10³)
g

Supplementary Fig. 4 | Properties of proteins detected by CF-MS.

a, PaxDb[8] consensus protein abundance of mouse and *Arabidopsis* proteins detected by CF-MS in the original meta-analysis or the updated resource, versus those not detected by CF-MS.

b, Relationship between PaxDb consensus protein abundance and the number of fractions in which a given protein was detected.

c, Relationship between PaxDb consensus protein abundance and the number of CF-MS experiments in which a given protein was detected.

d-e, Coverage of protein complexes from the CORUM database[9] in CFdb.

d, Cumulative distribution function showing the number of fractions in which each human protein from the CORUM protein complex database was quantified, in either the original meta-analysis (light grey) or CFdb (pink). Inset pie chart shows the total proportion of CORUM proteins that were detected in at least one fraction, shown separately for the original meta-analysis versus CFdb.

e, GO term enrichment among CORUM proteins detected only in the updated resource.

f, Saturation analysis showing the number of human, mouse, and *Arabidopsis* proteins ever detected by CF-MS when sampling experiments in random order from CFdb.

g, Projection of future increases in the number of human proteins ever detected by CF-MS after doubling the number of human CF-MS experiments in CFdb, based on data from **f**.

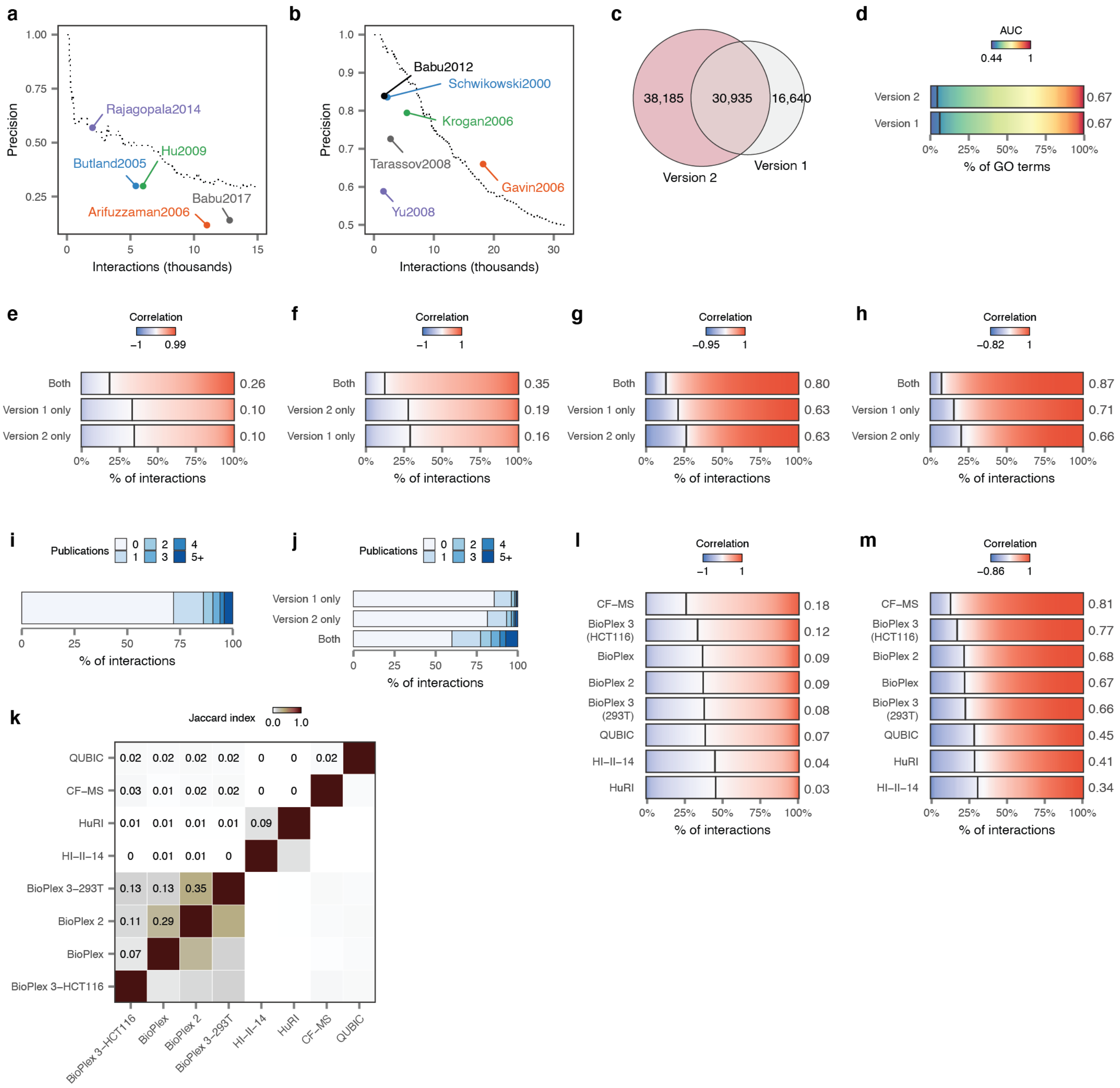

a
Precision
Interactions (thousands)
Rajagopala2014
Hu2009
Butland2005
Babu2017
Arifuzzaman2006
b
Babu2012
Schwikowski2000
Krogan2006
Tarassov2008
Gavin2006
Yu2008
c
38,185
30,935
16,640
Version 2
Version 1
d
AUC
Version 2 0.67
Version 1 0.67
% of GO terms
e
Correlation
Both 0.26
Version 1 only 0.10
Version 2 only 0.10
% of interactions
f
Both 0.35
Version 2 only 0.19
Version 1 only 0.16
g
Both 0.80
Version 1 only 0.63
Version 2 only 0.63
h
Both 0.87
Version 1 only 0.71
Version 2 only 0.66
i
Publications
j
Version 1 only
Version 2 only
Both
k
Jaccard index
QUBIC
CF–MS
HuRI
HI–II–14
BioPlex 3–293T
BioPlex 2
BioPlex
BioPlex 3–HCT116
l
CF–MS 0.18
BioPlex 3 (HCT116) 0.12
BioPlex 0.09
BioPlex 2 0.09
BioPlex 3 (293T) 0.08
QUBIC 0.07
HI–II–14 0.04
HuRI 0.03
m
CF–MS 0.81
BioPlex 3 (HCT116) 0.77
BioPlex 2 0.68
BioPlex 0.67
BioPlex 3 (293T) 0.66
QUBIC 0.45
HuRI 0.41
HI–II–14 0.34

Supplementary Fig. 5 | Interactome networks inferred by large-scale meta-analysis of CF-MS data.

a, Precision of the *E. coli* interactome inferred by meta-analysis of CF-MS experiments in CFdb for interaction networks of a given size, as compared to five high-throughput screens by Y2H or AP-MS[10–14].

b, Precision of the yeast interactome inferred by meta-analysis of CF-MS experiments in CFdb for interaction networks of a given size, as compared to six high-throughput screens by Y2H, AP-MS, or protein complementation assay and one network of literature-curated interactions[15–20].

c, Venn diagram showing overlap between the human CF-MS interactome described in our original meta-analysis[7] ("version 1") and the human interactome map presented in CFdb ("version 2").

d, Functional coherence of the human CF-MS interactome described in our original meta-analysis and the human interactome map presented in CFdb, as quantified by the AUC of protein function prediction in cross-validation[21].

e-f, Coexpression of interacting protein pairs present exclusively in human CF-MS interactome described in our original meta-analysis, the human interactome map presented in CFdb, or in both networks (**e,** coexpression in the ProteomeHD resource[22]; **f,** coexpression across a second proteomic dataset from cancer cell lines[23]). Text shows the median Pearson correlation. Vertical lines show the proportion of negatively correlated pairs[24].

g-h, As in **e-f,** but showing colocalization of interacting protein pairs by subcellular proteomics (**g,** SubCellBarCode[25]; **h,** LOPIT-DC[26]).

i, Overlap between the CFdb human interactome and literature-curated interactions. The number of publications supporting each protein-protein interaction is shown.

j, As in **i,** but shown separately for interactions found exclusively in human CF-MS interactome described in our original meta-analysis, the human interactome map presented in CFdb, or in both networks.

k, Overlap between high-throughput screens of the human interactome performed using Y2H or AP-MS since 2014, as quantified by the Jaccard index and as shown in **Supplementary Fig. 1** but here including the human interactome derived from meta-analysis of 166 CF-MS experiments.

l, Coexpression of interacting protein pairs in the CFdb human interactome, as compared to seven high-throughput screens of the human interactome, across a proteomic dataset from cancer cell lines[23].

m, Colocalization of interacting protein pairs in the CFdb human interactome, as compared to seven high-throughput screens of the human interactome, across a LOPIT-DC subcellular proteomics dataset[26].

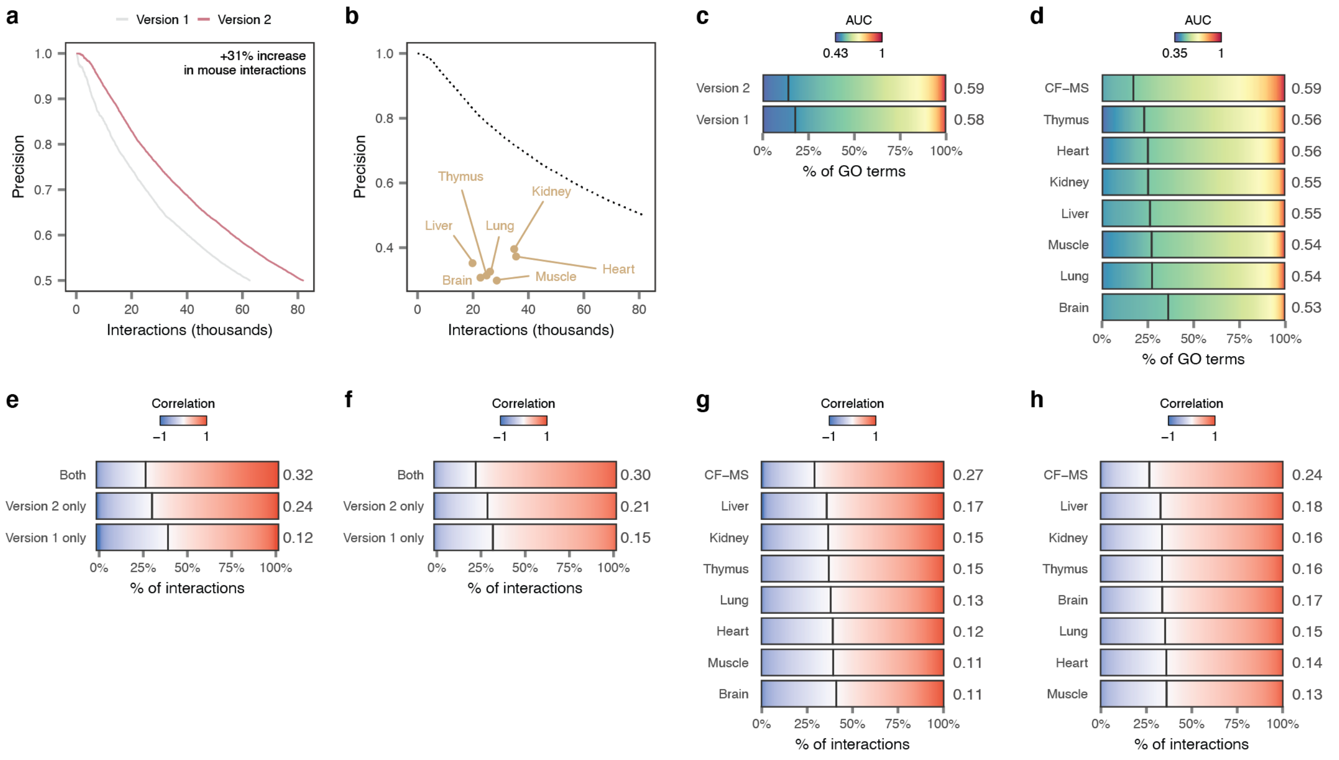

Supplementary Fig. 6 | Meta-analysis defines a consensus mouse interactome.

a, Precision of the mouse interactome inferred by meta-analysis of CF-MS experiments in CFdb as compared to our original meta-analysis, for interaction networks of a given size.

b, Precision of the mouse interactome inferred by meta-analysis of CF-MS experiments in CFdb for interaction networks of a given size, as compared to seven tissue-specific mouse networks previously inferred from PCP-SILAM data[27].

c, Functional coherence of mouse CF-MS interactomes inferred by meta-analysis of CF-MS datasets present in our original meta-analysis or in CFdb, as quantified by the AUC of protein function prediction in cross-validation[21].

d, Functional coherence of the CFdb mouse interactome, as compared to seven tissue-specific mouse networks previously inferred from PCP-SILAM data.

e-f, Coexpression of interacting protein pairs present in mouse CF-MS interactomes inferred by meta-analysis of CF-MS datasets present in our original meta-analysis, in CFdb, or in both networks, shown separately for two maps of the mouse tissue proteome (**e,** Geiger *et al.*[28]; **f,** Giansanti *et al.*[29]).

g-h, Coexpression of interacting protein pairs in the CFdb mouse interactome, as compared to seven tissue-specific mouse networks previously inferred from PCP-SILAM data, shown separately for two maps of the mouse tissue proteome (**g,** Geiger *et al.*[28]; **h,** Giansanti *et al.*[29]).

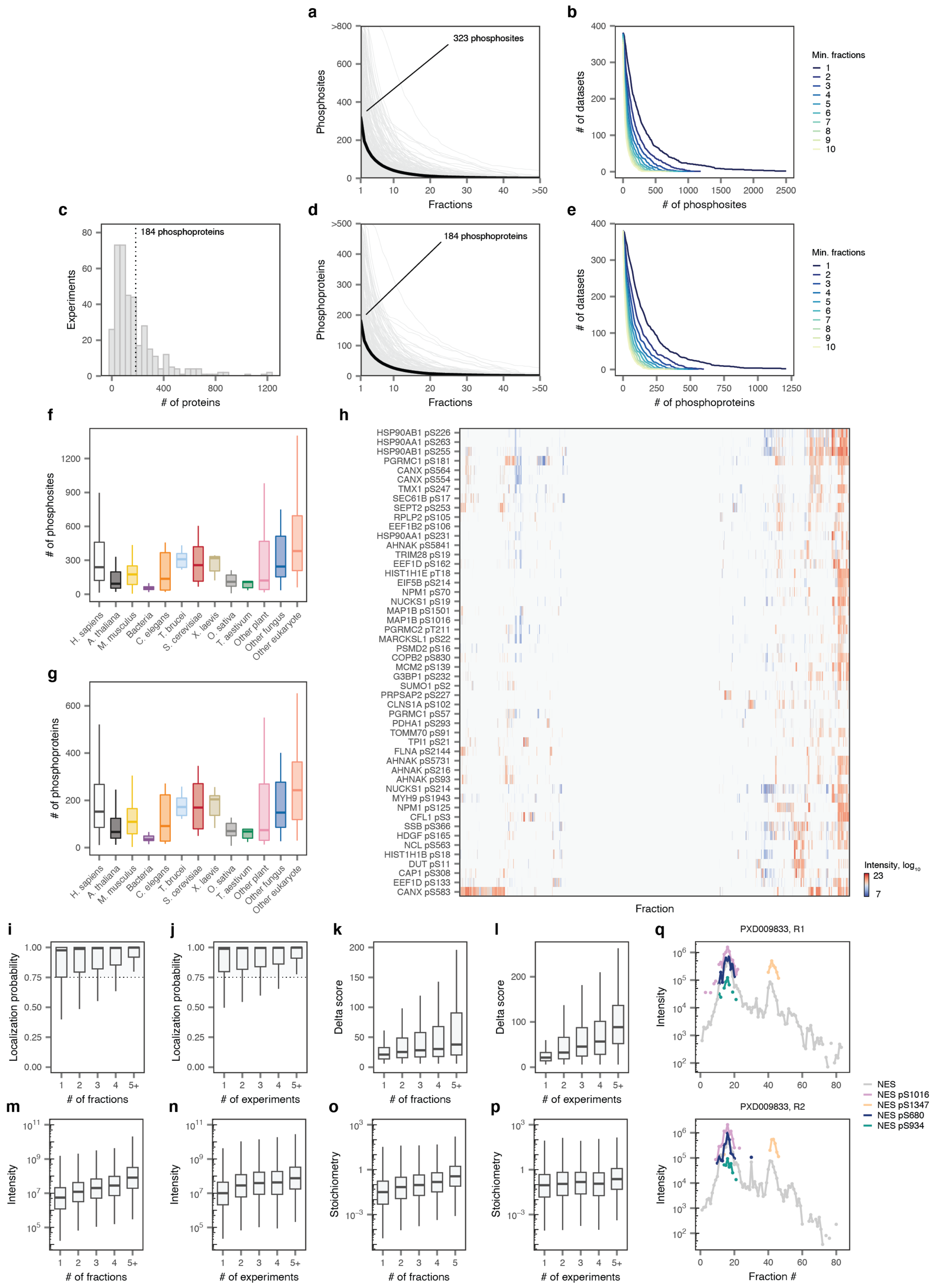

a
323 phosphosites
Phosphosites
Fractions
b
# of datasets
# of phosphosites
Min. fractions
c
184 phosphoproteins
Experiments
# of proteins
d
184 phosphoproteins
Phosphoproteins
Fractions
e
# of datasets
# of phosphoproteins
Min. fractions
f
# of phosphosites
H. sapiens
A. thaliana
M. musculus
Bacteria
C. elegans
T. brucei
S. cerevisiae
X. laevis
O. sativa
T. aestivum
Other plant
Other fungus
Other eukaryote
g
# of phosphoproteins
h
HSP90AB1 pS226
HSP90AA1 pS263
HSP90AB1 pS255
PGRMC1 pS181
CANX pS564
CANX pS554
TMX1 pS247
SEC61B pS17
SEPT2 pS253
RPLP2 pS105
EEF1B2 pS106
HSP90AA1 pS231
AHNAK pS5841
TRIM28 pS19
EEF1D pS162
HIST1H1E pT18
EIF5B pS214
NPM1 pS70
NUCKS1 pS19
MAP1B pS1501
MAP1B pS1016
PGRMC2 pT211
MARCKSL1 pS22
PSMD2 pS16
COPB2 pS830
MCM2 pS139
G3BP1 pS232
SUMO1 pS2
PRPSAP2 pS227
CLNS1A pS102
PGRMC1 pS57
PDHA1 pS293
TOMM70 pS91
TPI1 pS21
FLNA pS2144
AHNAK pS5731
AHNAK pS216
AHNAK pS93
NUCKS1 pS214
MYH9 pS1943
NPM1 pS125
CFL1 pS3
SSB pS366
HDGF pS165
NCL pS563
HIST1H1B pS18
DUT pS11
CAP1 pS308
EEF1D pS133
CANX pS583
Fraction
Intensity, log10
i
Localization probability
# of fractions
j
Localization probability
# of experiments
k
Delta score
# of fractions
l
Delta score
# of experiments
m
Intensity
# of fractions
n
Intensity
# of experiments
o
Stoichiometry
# of fractions
p
Stoichiometry
# of experiments
q
PXD009833, R1
PXD009833, R2
Intensity
Fraction #
NES
NES pS1016
NES pS1347
NES pS680
NES pS934

Supplementary Fig. 7 | Protein phosphorylation across 21,703 CF-MS fractions.

a, Number of phosphosites quantified in a minimum number of fractions in each CF-MS experiment (gray lines, individual datasets; blue line, mean across all datasets).
b, Cumulative distribution of the number of phosphosites quantified per experiment in at least one to ten fractions.
c, Histogram showing the number of phosphoproteins quantified in each CF-MS experiment.
d, As in **a,** but showing phosphoproteins instead of individual phosphosites.
e, As in **b,** but showing phosphoproteins instead of individual phosphosites.
f, Number of phosphosites quantified per experiment across major species or taxonomic groups in CFdb.
g, As in **f,** but showing phosphoproteins instead of individual phosphosites.
h, Clustered heatmap showing the abundance of the 50 most frequently quantified human phosphosites across 8,260 human CF-MS fractions.
i, Relationship between phosphosite localization probabilities and the number of fractions in which each phosphosite was quantified.
j, As in **i,** but showing experiments instead of fractions.
k, Relationship between phosphosite delta scores and the number of fractions in which each phosphosite was quantified.
l, As in **k,** but showing experiments instead of fractions.
m, Relationship between phosphosite intensities and the number of fractions in which each phosphosite was quantified.
n, As in **m,** but showing experiments instead of fractions.
o, Relationship between phosphosite stoichiometries and the number of fractions in which each phosphosite was quantified.
p, As in **o,** but showing experiments instead of fractions.
q, Examples of frequently quantified phosphosites in nestin. Chromatograms show the intensity of pS phosphopeptides (colors) or the parent protein (light grey). One phosphosite (pS1347) is specific to a low-intensity peak in the CF-MS data, whereas other phosphosites are detected exclusively in the highest-intensity peak (note that intensity is shown on a logarithmic scale).

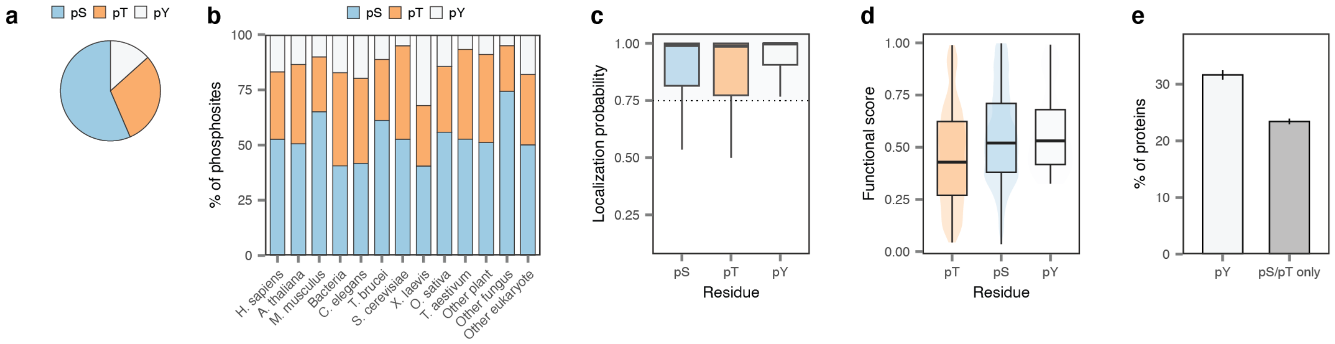

Supplementary Fig. 8 | Tyrosine phosphorylation in CFdb.

a, Proportion of phosphosites detected on serine, threonine, or tyrosine residues across all 411 CF-MS experiments in CFdb.

b, As in **a,** but showing proportions by major species or taxonomic groups in CFdb.

c, Localization probabilities of phosphosites detected on serine, threonine, or tyrosine residues, showing that phosphotyrosines are localized with comparable or higher probabilities as compared to phosphoserine or phosphothreonine residues.

d, Functional scores for phosphosites detected on serine, threonine, or tyrosine residues, showing that phosphotyrosines exhibit comparable or higher functional scores as compared to phosphoserine or phosphothreonine residues.

e, Phosphotyrosine-containing proteins in a meta-analysis of 6,801 phosphoproteomic experiments[30] are significantly enriched for proteins belonging to protein complexes, as compared to proteins phosphorylated only on serine or threonine residues ($p < 10^{-15}$, χ^2 test).

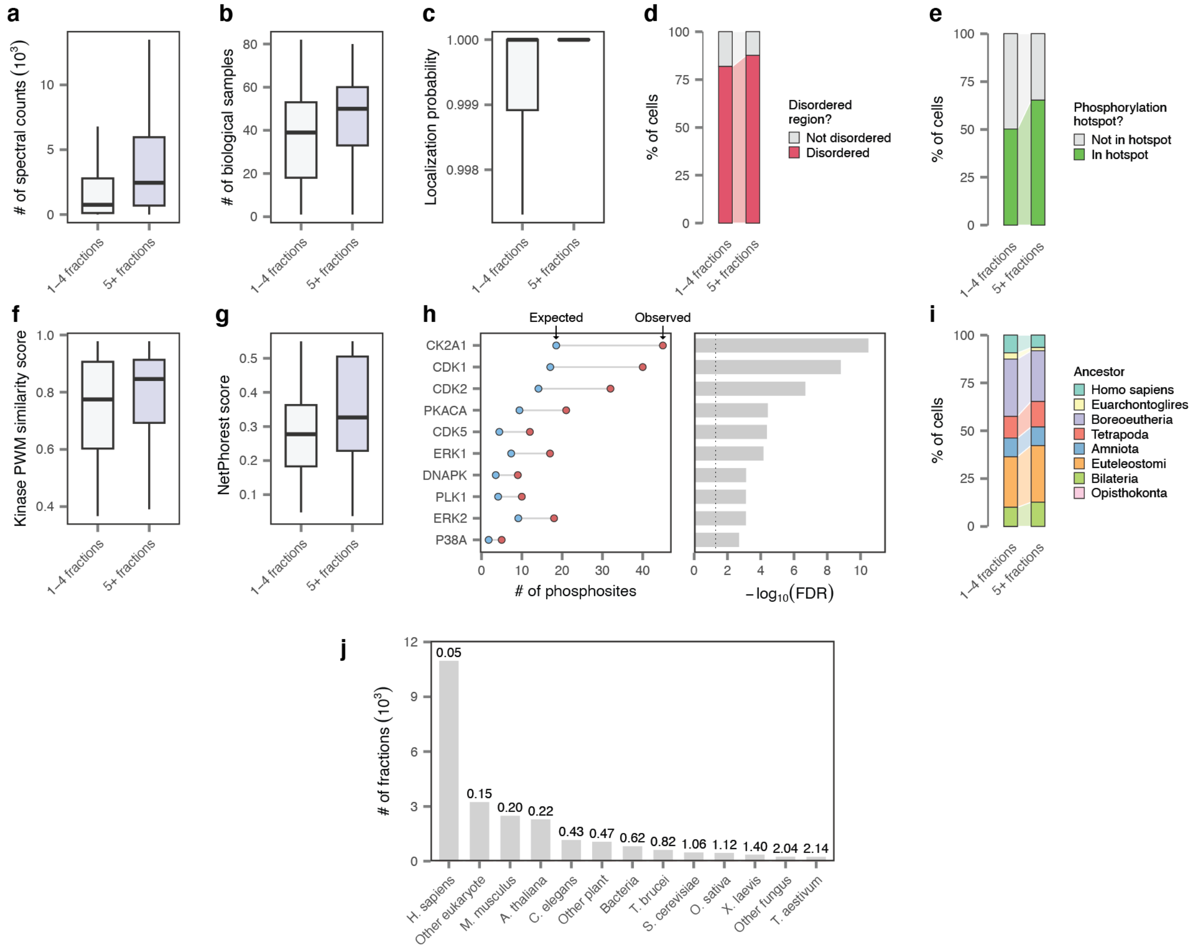

Supplementary Fig. 9 | Properties of frequently detected phosphosites.

a, Number of MS/MS spectra in which phosphosites were detected in an independent resource of 6,801 phosphoproteomics experiments, in which no samples overlapped with those used in CFdb[31], shown separately for phosphosites detected in 1-4 versus 5 or more CF-MS fractions ($p < 10^{-15}$, Wilcoxon rank-sum test).

b, As in **a,** but showing the number of biological samples in which phosphosites were detected ($p < 10^{-15}$, Wilcoxon rank-sum test).

c, As in **a,** but showing the maximum localization probability with which phosphosites were detected ($p < 10^{-15}$, Wilcoxon rank-sum test).

d, Proportion of phosphosites located within predicted intrinsically disordered regions, shown separately for phosphosites detected in 1-4 versus 5 or more CF-MS fractions ($p = 2.1 \times 10^{-15}$, χ^2 test).

e, As in **d,** but showing phosphosites located within phosphorylation hotspots ($p = 2.0 \times 10^{-8}$, χ^2 test).

f, As in **a,** but showing the maximum kinase position weight motif matrix similarity score for each phosphosite ($p < 10^{-15}$, Wilcoxon rank-sum test).

g, As in **a,** but showing the maximum NetPhorest posterior probability for each phosphosite ($p < 10^{-15}$, Wilcoxon rank-sum test).

h, Kinase-substrate enrichment analysis of phosphosites. Left, number of expected (blue) versus observed (red) substrates of kinases from the PhosphoSitePlus database among phosphosites detected in 5 or more CF-MS fractions, compared to phosphosites ever detected by CF-MS; right, statistical significance of the observed enrichment.

i, As in **d,** but showing the inferred ancestral age of each phosphorylation site ($p = 1.3 \times 10^{-15}$, χ^2 test).

j, Bars, total number of CF-MS fractions collected per major species or taxonomic groups. Text, proportion of fractions per species corresponding to detection in five or more CF-MS fractions.

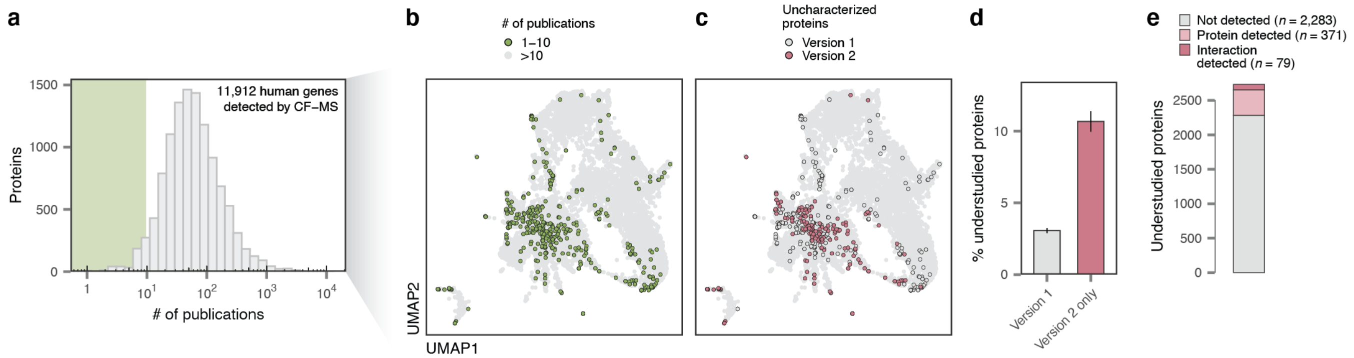

Supplementary Fig. 10 | Understudied proteins in CFdb.

a, Number of publications linked to each human protein quantified in CFdb.

b-c, UMAP visualizations of protein-protein co-association patterns across 166 human CF-MS experiments (**b,** highlighting proteins linked to less than 10 publications (‘understudied’ proteins); **c,** highlighting understudied proteins detected only in CFdb).

d, Proportion of understudied proteins detected in the original meta-analysis (“version 1”) or exclusively in CFdb ($p < 10^{-15}$, χ^2 test).

e, Overview of understudied human proteins for which an interaction was detected or the protein was detected in CFdb.

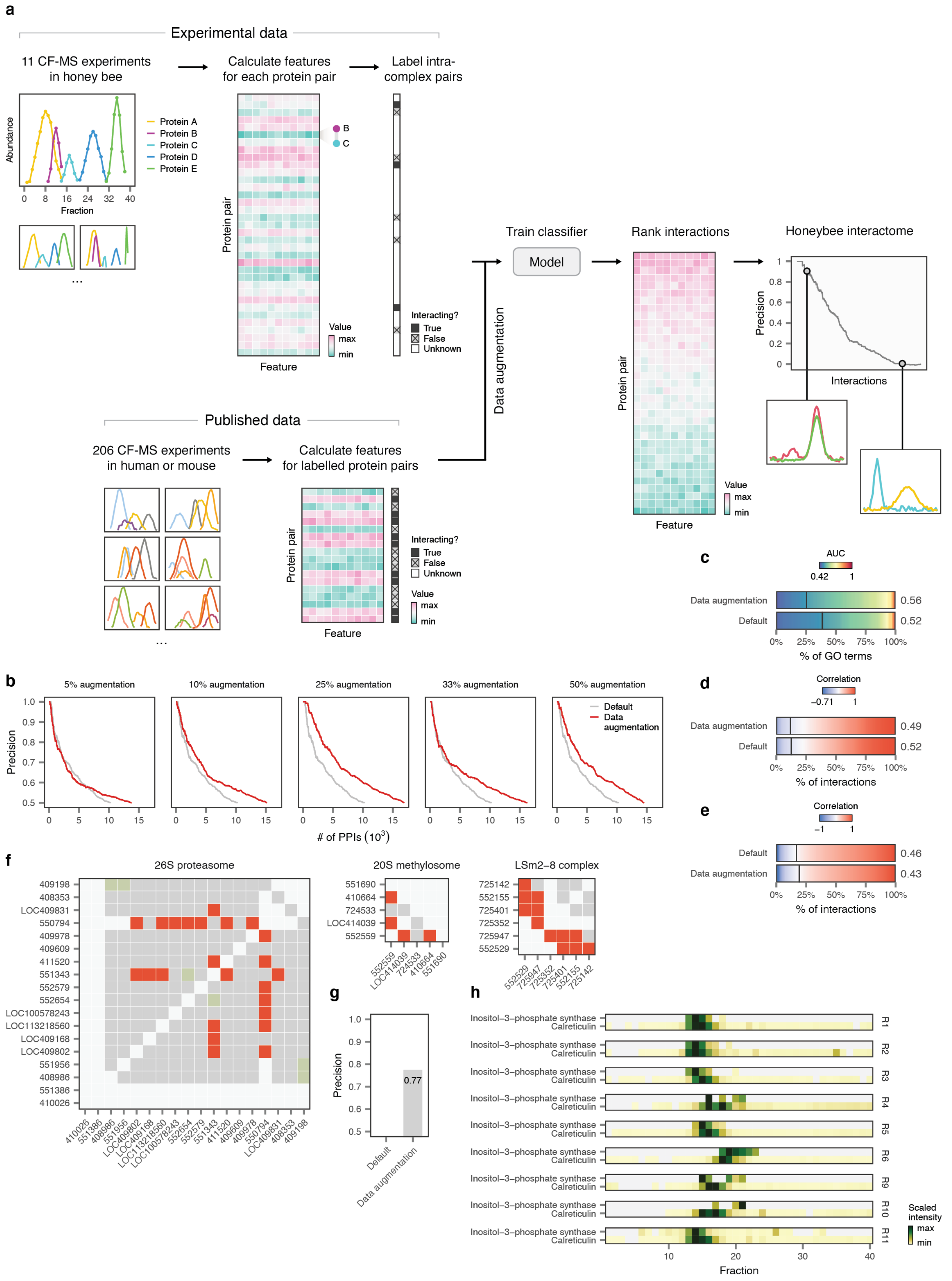
a
Experimental data
11 CF-MS experiments in honey bee
Calculate features for each protein pair
Label intra-complex pairs
Abundance
Fraction
Protein A
Protein B
Protein C
Protein D
Protein E
Protein pair
Feature
Value
max
min
Interacting?
True
False
Unknown
Published data
206 CF-MS experiments in human or mouse
Calculate features for labelled protein pairs
Data augmentation
Train classifier
Model
Rank interactions
Honeybee interactome
Precision
Interactions
b
5% augmentation
10% augmentation
25% augmentation
33% augmentation
50% augmentation
Default
Data augmentation
# of PPIs (10³)
c
AUC
% of GO terms
0.56
0.52
d
Correlation
% of interactions
0.49
0.52
e
0.46
0.43
f
26S proteasome
20S methylosome
LSm2–8 complex
g
0.77
h
Inositol–3–phosphate synthase
Calreticulin
Scaled intensity
Fraction

Supplementary Fig. 11 | Mapping the honey bee interactome with data augmentation using CFdb.
a, Schematic overview of a data augmentation strategy in which published CF-MS data from CFdb is leveraged at a massive scale to interpret experiments done in individual laboratories.
b, Functional coherence of the honey bee CF-MS interactome mapped with or without data augmentation on CFdb.
c, Precision of the honey bee CF-MS interactome for networks of a given size, with varying proportions of external features incorporated in the training procedure.
d, Coexpression of interacting protein pairs in the honey bee CF-MS interactome mapped with or without data augmentation.
e, Co-fractionation of *Drosophila* orthologs of interacting protein pairs in the honey bee CF-MS interactome mapped with or without data augmentation.
f, Examples of CORUM protein complexes with one-to-one orthologs in honey bee with intra-complex interactions better resolved by network inference after data augmentation.
g, Precision with which the putative interaction between calreticulin and inositol-3-phosphate synthase was recovered the honey bee CF-MS interactome mapped with or without data augmentation.
h, Elution profiles of calreticulin and inositol-3-phosphate synthase in nine CF-MS replicates in which both proteins were quantified.

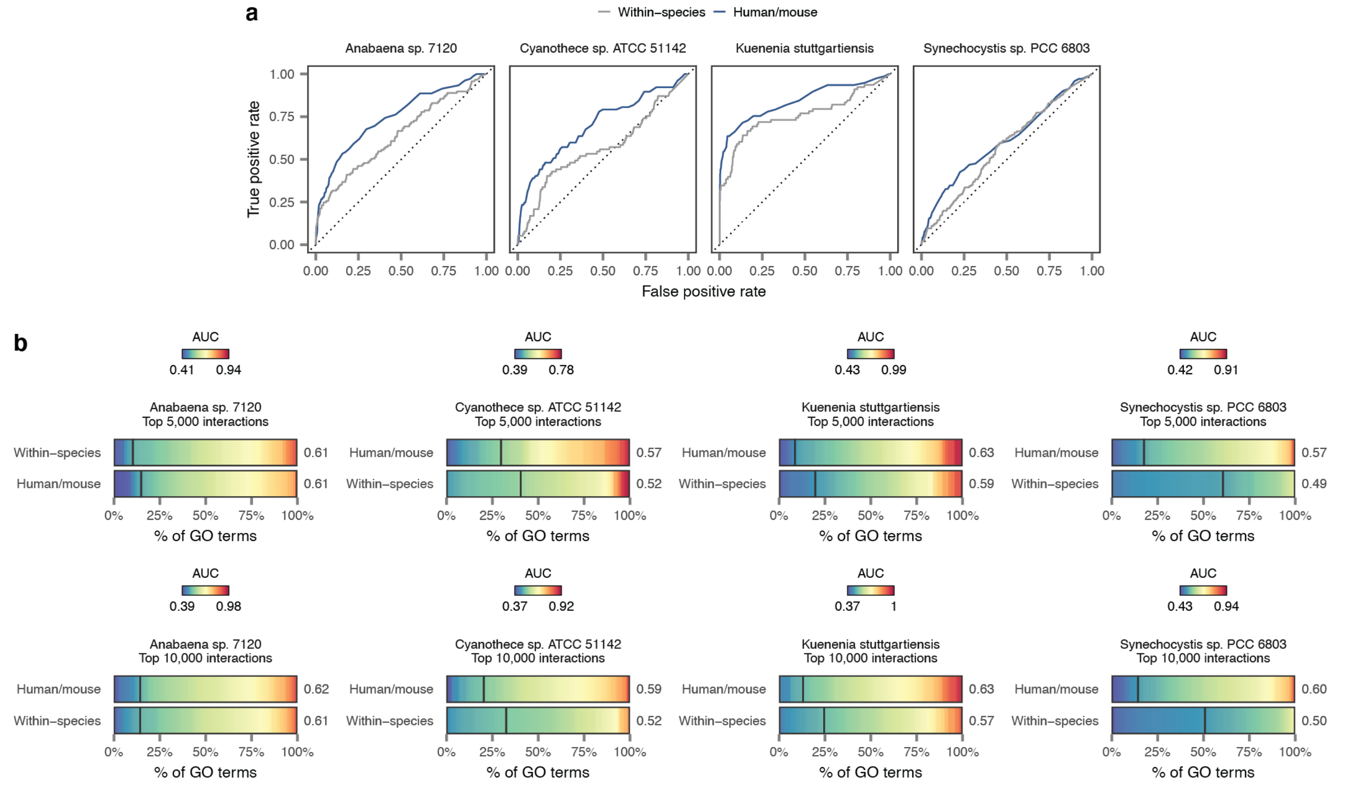

Supplementary Fig. 12 | Interactome mapping in understudied prokaryotes without a training set of known protein complexes.
a, Receiver operating characteristic (ROC) curves demonstrating separation of intra- and inter-complex interactions by random forest classifiers trained in cross-validation on species-specific protein complexes ("within-species") versus on 206 human and mouse CF-MS experiments ("human/mouse").
b, Functional coherence of protein interaction networks for four understudied prokaryotes reconstructed by random forest classifiers trained in cross-validation on species-specific protein complexes ("within-species") versus on 206 human and mouse CF-MS experiments ("human/mouse"), shown separately for interaction networks at two fixed sizes (top-5,000 versus top-10,000 interactions).

Supplementary Table 1 | DIA windows

#MS Type	Cycle Id	Start IM [1/K0]	End IM [1/K0]	Start Mass [m/z]	End Mass [m/z]	CE [eV]
MS1	0	-	-	-	-	-
PASEF	1	0.9614	1.2687	914.5	949.5	-
PASEF	1	0.7	0.9427	319.5	354.5	-
PASEF	2	0.9806	1.2879	949.5	984.5	-
PASEF	2	0.7	0.9618	354.5	389.5	-
PASEF	3	0.9998	1.3071	984.5	1019.5	-
PASEF	3	0.7	0.981	389.5	424.5	-
PASEF	4	1.0189	1.3262	1019.5	1054.5	-
PASEF	4	0.7	1.0002	424.5	459.5	-
PASEF	5	1.0381	1.3454	1054.5	1089.5	-
PASEF	5	0.7121	1.0194	459.5	494.5	-
PASEF	6	0.7312	1.0385	494.5	529.5	-
PASEF	7	0.7504	1.0577	529.5	564.5	-
PASEF	8	0.7696	1.0769	564.5	599.5	-
PASEF	9	0.7888	1.0961	599.5	634.5	-
PASEF	10	0.808	1.1153	634.5	669.5	-
PASEF	11	0.8271	1.1344	669.5	704.5	-
PASEF	12	0.8463	1.1536	704.5	739.5	-
PASEF	13	0.8655	1.1728	739.5	774.5	-
PASEF	14	0.8847	1.192	774.5	809.5	-
PASEF	15	0.9039	1.2112	809.5	844.5	-
PASEF	16	0.923	1.2303	844.5	879.5	-
PASEF	17	0.9422	1.2495	879.5	914.5	-

Supplementary Note

Overview of machine-learning approach. To reconstruct protein interaction networks from CF-MS data, we applied a supervised machine learning approach similar to that implemented in a number of published studies and software tools[7,27,32–42]. Briefly, for each set of CF-MS experiments, a series of all-by-all pairwise scores were calculated between the elution profiles of all quantified proteins. The calculated pairwise scores were then merged across CF-MS experiments to produce a single feature matrix, which was provided to a random forest classifier as input alongside a set of known protein complexes from CORUM[9]. A pair of proteins was labeled "positive" if both proteins were in the same CORUM complex, and "negative" if proteins were both in the set of CORUM complexes, but not part of the same complex. The classifier was trained to distinguish positive and negative examples (that is, interacting versus non-interacting protein pairs). Classifier training was performed in ten-fold cross-validation, both to minimize overfitting and to allow the classifier to make predictions for protein pairs within the training set of known protein complexes without data leakage. Protein pairs were ranked in descending order by their mean classifier score across all ten folds. Finally, the classifier score calculated for each protein pair in the ranked list was then converted to a measure of precision for each interaction by calculating the ratio of true positives to true positives plus true negatives among interactions assigned that score or greater by the classifier. Below, we elaborate on each step in this workflow.

Data preprocessing and quality control. MaxQuant searches were carried out as described in the Methods, and proteinGroups files were preprocessed by removing potential contaminants, reverse hits and proteins identified only by peptides carrying one or more modified amino acids, and mapping protein groups to gene symbols to enable matching across replicates. Proteins quantified in less than four fractions per dataset were filtered. Protein abundance was quantified using the iBAQ algorithm[43] for label-free datasets, the isotopologue ratio for SILAC and dimethyl datasets, and the corrected reporter intensity for TMT datasets. These steps afforded, for each CF-MS experiment, a protein abundance matrix A of dimensions $I \times J$ where I is the number of proteins quantified, J is the number of fractions profiled by CF-MS, and $A_{i,j}$ represents the abundance of protein i in fraction j.

Feature calculation. Proteins that physically interact as part of the same protein complex are expected to co-elute across a biochemical gradient. To quantify the similarity of any given pair of proteins, we next calculated a series of all-by-all pairwise scores between each of the protein elution curves in any given CF-MS experiment. This yielded, for each protein abundance matrix $A_{I \times J}$, a feature matrix F of dimensions $P \times N$, where $P = (I \times (I-1)/2)$ is the number of protein pairs being scored, N is the number of features calculated for each protein pair, and $F_{p,n}$ reflects the value of feature f for protein pair p.

The specific number of features that were calculated for each protein pair varied across species based on the number of CF-MS experiments that were being meta-analyzed in that species, as described in the main text (Methods). Up to four features were calculated for each protein pair, these being:

- The distance correlation between protein elution profiles, calculated using the 'Pigengene' R package.
- The weighted cross-correlation between protein elution profiles, calculated using the 'wccsom' R package.
- The cosine similarity between protein elution profiles, calculated using the 'lsa' R package.
- The mutual information between (discretized) protein elution profiles, calculated using the 'WGCNA' R package.

Additional preprocessing steps were applied to the protein abundance matrix A depending on the specific feature being calculated, also as described in the main text (Methods). Specifically, the distance correlation and weighted cross-correlation were calculated between protein abundance profiles in which missing values were replaced with zeroes, whereas in the calculation of the mutual information, missing values were not imputed. The cosine similarity was calculated between protein abundance profiles in which missing values were replaced with near-zero noise sampled from a uniform distribution $U(0, 0.001)$[7,44].

Feature matrices were then merged across CF-MS experiments to create a single master feature matrix G for each species of dimensions $Q \times N$, where Q is the total number of protein pairs detected in at least one CF-MS experiment. In human, for example, G consisted of a matrix with 47,455,274 rows (protein pairs) and 166 columns (features; only a single feature was calculated for each experiment in the human data, as described in the main text). This matrix contained missing values when one or both of the proteins in a given protein pair were not quantified in a particular CF-MS experiment. Missing values in the feature matrix were imputed with the median value of that feature[7].

Labels. A gold standard reference set of positive and negative interactions was generated from the CORUM database of curated mammalian protein complexes. Positive examples were defined as pairs of proteins that are part of the same complex ("intra-complex" interactions), whereas negative examples were defined as pairs of proteins that are both in the set of complexes but not part of the same complex ("inter-complex" interactions). Pairs of proteins in which one or both proteins were not part of a CORUM complex were left unlabelled. Protein complexes from CORUM were mapped to their orthologs in each species using InParanoid[45]. The feature matrix G was then subset to include only labelled protein pairs to give the labelled feature matrix X.

Cross-validation. Given the feature matrix X and label vector y, we next proceeded to train random forest classifiers in ten-fold cross-validation using the R package 'randomForest.' We performed cross-validation by splitting the dataset into ten folds and withheld each of these folds in turn. A random forest model was then trained on the other nine folds. The trained model was then applied to predict the probability of each protein pair being a positive example, both for the held-out fold and for all unlabeled protein pairs.

Protein pairs were ranked in descending order by their mean classifier score across all ten folds. At each point in the resulting ranked list n, the precision of the network up to that protein pair was calculated as

$$Precision = \frac{TP_n}{TP_n + FP_n}$$

where TP_n and FP_n are the number of true positives and false positives among the n top-ranked protein pairs, respectively.

Supplementary References

1. Rolland, T. *et al.* A proteome-scale map of the human interactome network. *Cell* **159,** 1212–1226 (2014).
2. Hein, M. Y. *et al.* A human interactome in three quantitative dimensions organized by stoichiometries and abundances. *Cell* **163,** 712–723 (2015).
3. Huttlin, E. L. *et al.* The BioPlex network: a systematic exploration of the human interactome. *Cell* **162,** 425–440 (2015).
4. Huttlin, E. L. *et al.* Architecture of the human interactome defines protein communities and disease networks. *Nature* **545,** 505–509 (2017).
5. Luck, K. *et al.* A reference map of the human binary protein interactome. *Nature* **580,** 402–408 (2020).
6. Huttlin, E. L. *et al.* Dual proteome-scale networks reveal cell-specific remodeling of the human interactome. *Cell* **184,** 3022–3040 (2021).
7. Skinnider, M. A. & Foster, L. J. Meta-analysis defines principles for the design and analysis of co-fractionation mass spectrometry experiments. *Nat. Methods* **18,** 806–815 (2021).
8. Wang, M., Herrmann, C. J., Simonovic, M., Szklarczyk, D. & von Mering, C. Version 4.0 of PaxDb: protein abundance data, integrated across model organisms, tissues, and cell-lines. *Proteomics* **15,** 3163–3168 (2015).
9. Tsitsiridis, G. *et al.* CORUM: the comprehensive resource of mammalian protein complexes–2022. *Nucleic Acids Res.* **51,** D539–D545 (2023).
10. Butland, G. *et al.* Interaction network containing conserved and essential protein complexes in Escherichia coli. *Nature* **433,** 531–537 (2005).
11. Arifuzzaman, M. *et al.* Large-scale identification of protein–protein interaction of Escherichia coli K-12. *Genome Res.* **16,** 686–691 (2006).
12. Hu, P. *et al.* Global functional atlas of Escherichia coli encompassing previously uncharacterized proteins. *PLoS Biol.* **7,** e1000096 (2009).
13. Rajagopala, S. V. *et al.* The binary protein-protein interaction landscape of Escherichia coli. *Nat. Biotechnol.* **32,** 285–290 (2014).
14. Babu, M. *et al.* Global landscape of cell envelope protein complexes in Escherichia coli. *Nat. Biotechnol.* **36,** 103–112 (2018).
15. Schwikowski, B., Uetz, P. & Fields, S. A network of protein-protein interactions in yeast. *Nat. Biotechnol.* **18,** 1257–1261 (2000).
16. Gavin, A.-C. *et al.* Proteome survey reveals modularity of the yeast cell machinery. *Nature* **440,** 631–636 (2006).
17. Krogan, N. J. *et al.* Global landscape of protein complexes in the yeast Saccharomyces cerevisiae. *Nature* **440,** 637–643 (2006).
18. Tarassov, K. *et al.* An in vivo map of the yeast protein interactome. *Science* **320,** 1465–1470 (2008).
19. Yu, H. *et al.* High-quality binary protein interaction map of the yeast interactome network. *Science* **322,** 104–110 (2008).
20. Babu, M. *et al.* Interaction landscape of membrane-protein complexes in Saccharomyces cerevisiae. *Nature* **489,** 585–589 (2012).
21. Ballouz, S., Weber, M., Pavlidis, P. & Gillis, J. EGAD: ultra-fast functional analysis of gene networks. *Bioinformatics* **33,** 612–614 (2017).
22. Kustatscher, G. *et al.* Co-regulation map of the human proteome enables identification of protein functions. *Nat. Biotechnol.* **37,** 1361–1371 (2019).
23. Lapek Jr, J. D. *et al.* Detection of dysregulated protein-association networks by high-throughput proteomics predicts cancer vulnerabilities. *Nat. Biotechnol.* **35,** 983–989 (2017).
24. Stacey, R. G., Skinnider, M. A., Chik, J. H. & Foster, L. J. Context-specific interactions in literature-curated protein interaction databases. *BMC Genomics* **19,** 1–10 (2018).
25. Orre, L. M. *et al.* SubCellBarCode: proteome-wide mapping of protein localization and relocalization. *Mol. Cell* **73,** 166–182 (2019).
26. Geladaki, A. *et al.* Combining LOPIT with differential ultracentrifugation for high-resolution spatial proteomics. *Nat. Commun.* **10,** 331 (2019).
27. Skinnider, M. A. *et al.* An atlas of protein-protein interactions across mouse tissues. *Cell* **184,** 4073–4089 (2021).
28. Geiger, T. *et al.* Initial quantitative proteomic map of 28 mouse tissues using the SILAC mouse. *Mol. Cell. Proteomics* **12,** 1709–1722 (2013).
29. Giansanti, P. *et al.* Mass spectrometry-based draft of the mouse proteome. *Nat. Methods* **19,** 803–811 (2022).
30. Ochoa, D. *et al.* The functional landscape of the human phosphoproteome. *Nat. Biotechnol.* **38,** 365–373 (2020).
31. Ochoa, D. *et al.* An atlas of human kinase regulation. *Mol. Syst. Biol.* **12,** 888 (2016).
32. Havugimana, P. C. *et al.* A census of human soluble protein complexes. *Cell* **150,** 1068–1081 (2012).
33. Wan, C. *et al.* Panorama of ancient metazoan macromolecular complexes. *Nature* **525,** 339–344 (2015).
34. Kerr, C. H. *et al.* Dynamic rewiring of the human interactome by interferon signaling. *Genome Biol.* **21,** 1–36 (2020).
35. McWhite, C. D. *et al.* A pan-plant protein complex map reveals deep conservation and novel assemblies. *Cell* **181,** 460–474 (2020).
36. Hu, L. Z. *et al.* EPIC: software toolkit for elution profile-based inference of protein complexes. *Nat. Methods* **16,** 737–742 (2019).
37. Pourhaghighi, R. *et al.* BrainMap elucidates the macromolecular connectivity landscape of mammalian brain. *Cell Syst.* **11,** 208 (2020).
38. Kastritis, P. L. *et al.* Capturing protein communities by structural proteomics in a thermophilic eukaryote. *Mol. Syst. Biol.* **13,** 936 (2017).
39. Larance, M. *et al.* Global membrane protein interactome analysis using in vivo crosslinking and mass spectrometry-based protein correlation profiling. *Mol. Cell. Proteomics* **15,** 2476–2490 (2016).
40. Crozier, T. W. M., Tinti, M., Larance, M., Lamond, A. I. & Ferguson, M. A. J. Prediction of protein complexes in Trypanosoma brucei by protein correlation profiling mass spectrometry and machine learning. *Mol. Cell. Proteomics* **16,** 2254–2267 (2017).
41. Hillier, C. *et al.* Landscape of the Plasmodium interactome reveals both conserved and species-specific functionality. *Cell Rep.* **28,** 1635–1647 (2019).
42. Liebeskind, B. J., Aldrich, R. W. & Marcotte, E. M. Ancestral reconstruction of protein interaction networks. *PLoS Comput. Biol.* **15,** e1007396 (2019).
43. Schwanhäusser, B. *et al.* Global quantification of mammalian gene expression control. *Nature* **473,** 337–342 (2011).
44. Skinnider, M. A., Cai, C., Stacey, R. G. & Foster, L. J. PrInCE: an R/Bioconductor package for protein–protein interaction network inference from co-fractionation mass spectrometry data. *Bioinformatics* **37,** 2775–2777 (2021).
45. Sonnhammer, E. L. & Östlund, G. InParanoid 8: orthology analysis between 273 proteomes, mostly eukaryotic. *Nucleic Acids Res.* **43,** D234–D239 (2015).
