# Supplementary material for: Mapping protein states and interactions across the tree of life with co-fractionation mass spectrometry
Source: Nat Commun. 2023 Dec 15;14:8365. doi: 10.1038/s41467-023-44139-5 (PMC10724252; doi:10.1038/s41467-023-44139-5)
Supplement: Supplementary file 3 — Description of Additional Supplementary Files [file 41467_2023_44139_MOESM3_ESM.pdf]

# <u>Description of Additional Supplementary Files</u>

**Supplementary Data 1**
Complete list of all CF-MS experiments, **a,** and raw mass spectrometry files, **b,** analyzed in this study.

**Supplementary Data 2**
Number of fractions in which each protein across all 32 species was quantified in CFdb.

**Supplementary Data 3**
Interactome networks for human, **a,** and mouse, **b.**

**Supplementary Data 4**
Phosphosites prioritized by CF-MS data integration across 31 non-human species.

**Supplementary Data 5**
Honey bee interactome networks inferred with, **a,** and without, **b,** data augmentation.
