# Supplementary material for: Mapping protein states and interactions across the tree of life with co-fractionation mass spectrometry
Source: Nat Commun. 2023 Dec 15;14:8365. doi: 10.1038/s41467-023-44139-5 (PMC10724252; doi:10.1038/s41467-023-44139-5)
Supplement: Supplementary file 9 — Reporting Summary [file 41467_2023_44139_MOESM9_ESM.pdf]

nature portfolio

Corresponding author(s): Leonard Foster

Last updated by author(s): Nov 24, 2023

Reporting Summary

Nature Portfolio wishes to improve the reproducibility of the work that we publish. This form provides structure for consistency and transparency in reporting. For further information on Nature Portfolio policies, see our Editorial Policies and the Editorial Policy Checklist.

Statistics

For all statistical analyses, confirm that the following items are present in the figure legend, table legend, main text, or Methods section.

n/a	Confirmed	
☐	☒	The exact sample size (*n*) for each experimental group/condition, given as a discrete number and unit of measurement
☐	☒	A statement on whether measurements were taken from distinct samples or whether the same sample was measured repeatedly
☐	☒	The statistical test(s) used AND whether they are one- or two-sided<br>*Only common tests should be described solely by name; describe more complex techniques in the Methods section.*
☒	☐	A description of all covariates tested
☒	☐	A description of any assumptions or corrections, such as tests of normality and adjustment for multiple comparisons
☐	☒	A full description of the statistical parameters including central tendency (e.g. means) or other basic estimates (e.g. regression coefficient) AND variation (e.g. standard deviation) or associated estimates of uncertainty (e.g. confidence intervals)
☐	☒	For null hypothesis testing, the test statistic (e.g. F, t, r) with confidence intervals, effect sizes, degrees of freedom and P value noted<br>*Give P values as exact values whenever suitable.*
☒	☐	For Bayesian analysis, information on the choice of priors and Markov chain Monte Carlo settings
☒	☐	For hierarchical and complex designs, identification of the appropriate level for tests and full reporting of outcomes
☐	☒	Estimates of effect sizes (e.g. Cohen's d, Pearson's r), indicating how they were calculated

Our web collection on statistics for biologists contains articles on many of the points above.

Software and code

Policy information about availability of computer code

Data collection	The raw data re-analyzed in this study was obtained from publicly available proteomics repositories (PRIDE and MASSIVE) and therefore did not require software to collect. Proteomics data collected in-house did not require software to collect.
Data analysis	Raw proteomics data from publicly available repositories were re-analyzed with MaxQuant (version 1.6.5.0) and RawTools (version 2.0.2). Data analysis was performed in R (version 4.2.2). Custom code is available from the following repositories: source code used to download and re-analyze publicly available CF-MS data using MaxQuant is available at https://github.com/skinnider/CFdb-searches. Source code used to carry out the analyses presented in the paper, with relevant intermediate data files, is available from https://github.com/skinnider/CFdb-analysis. The CFTK R package is available from https://github.com/fosterlab/CFTK. Proteomics data generated in-house was analyzed with DIA-NN (version 1.8.1).

For manuscripts utilizing custom algorithms or software that are central to the research but not yet described in published literature, software must be made available to editors and reviewers. We strongly encourage code deposition in a community repository (e.g. GitHub). See the Nature Portfolio guidelines for submitting code & software for further information.

Data

Policy information about availability of data

All manuscripts must include a data availability statement. This statement should provide the following information, where applicable:

- Accession codes, unique identifiers, or web links for publicly available datasets
- A description of any restrictions on data availability
- For clinical datasets or third party data, please ensure that the statement adheres to our policy

A list of all raw mass spectrometry files analyzed in this study and their accession numbers in PRIDE or MassIVE repositories is provided in Supplementary Data 1. Source code used to download and re-analyzed published CF-MS data is available at https://github.com/skinnider/CFdb-searches. Processed chromatograms, phosphosite chromatograms, and MaxQuant 'proteinGroups.txt' and 'Phospho(STY) sites.txt' files are available via Zenodo at https://doi.org/10.5281/zenodo.8008094. Pre-calculated features for each species are available via Zenodo at https://doi.org/10.5281/zenodo.8005773. Predicted interactomes for each species are available via Zenodo at https://doi.org/10.5281/zenodo.10038713. Complete MaxQuant outputs for all 411 CF-MS experiments have been deposited to the PRIDE repository87 with the dataset identifier PXD042664 [http://proteomecentral.proteomexchange.org/cgi/GetDataset?ID=PXD042664]. Honey bee CF-MS datasets have been deposited to the PRIDE repository with the dataset identifier PXD042820 [http://proteomecentral.proteomexchange.org/cgi/GetDataset?ID=PXD042820]. Other databases used in the study were as follows: PaxDb (https://pax-db.org/), Human Protein Atlas (https://www.proteinatlas.org/), CORUM (http://mips.helmholtz-muenchen.de/corum/), EcoCyc (https://ecocyc.org/), PhosphoSitePlus (https://www.phosphosite.org/homeAction.action), and BioGRID (https://thebiogrid.org/). Source data are provided with this paper.

Research involving human participants, their data, or biological material

Policy information about studies with human participants or human data. See also policy information about sex, gender (identity/presentation), and sexual orientation and race, ethnicity and racism.

Reporting on sex and gender	N/A
Reporting on race, ethnicity, or other socially relevant groupings	N/A
Population characteristics	N/A
Recruitment	N/A
Ethics oversight	N/A

Note that full information on the approval of the study protocol must also be provided in the manuscript.

Field-specific reporting

Please select the one below that is the best fit for your research. If you are not sure, read the appropriate sections before making your selection.

☒ Life sciences ☐ Behavioural & social sciences ☐ Ecological, evolutionary & environmental sciences

For a reference copy of the document with all sections, see nature.com/documents/nr-reporting-summary-flat.pdf

Life sciences study design

All studies must disclose on these points even when the disclosure is negative.

Sample size	The sample size was determined by reviewing the literature to assemble a list of all published studies with data available on public proteomics repositories. Sample sizes used in individual experiments re-analyzed in this study were chosen at the discretion of the original investigators.
Data exclusions	Published CF-MS data collected using data-independent acquisition (DIA) was excluded as it could not be re-analyzed using a consistent workflow with the vast majority of published CF-MS data.
Replication	Replication was performed in the sense that the conclusions are based on a meta-analysis of essentially all published experiments with publicly available data at the time of analysis. Individual experiments re-analyzed in this study may or may not have performed biological replicates at the discretion of the original investigators.
Randomization	Randomization was not relevant to the study because it focused on the re-analysis of published data and consequently randomization was not possible given that all data had already been generated.
Blinding	Blinding was not relevant to the study because it focused on the re-analysis of published data and consequently blinding was not possible given that all data had already been generated.

Reporting for specific materials, systems and methods

We require information from authors about some types of materials, experimental systems and methods used in many studies. Here, indicate whether each material, system or method listed is relevant to your study. If you are not sure if a list item applies to your research, read the appropriate section before selecting a response.

Materials & experimental systems

n/a	Involved in the study
☒	☐ Antibodies
☒	☐ Eukaryotic cell lines
☒	☐ Palaeontology and archaeology
☐	☒ Animals and other organisms
☒	☐ Clinical data
☒	☐ Dual use research of concern
☒	☐ Plants

Methods

n/a	Involved in the study
☒	☐ ChIP-seq
☒	☐ Flow cytometry
☒	☐ MRI-based neuroimaging

Animals and other research organisms

Policy information about studies involving animals; ARRIVE guidelines recommended for reporting animal research, and Sex and Gender in Research

Laboratory animals	The study did not involve laboratory animals.
Wild animals	The study did not involve wild animals.
Reporting on sex	All worker bees are female, and consequently the study involved only female wild animals.
Field-collected samples	The study involved honey bees (Apis mellifera), which were maintained at the University of British Columbia farm apiary with maintenance by regular beekeeping practices, and whose ages were not determined precisely.
Ethics oversight	No ethics oversight was required as the University of British Columbia does not require institutional review for experiments involving honey bees.

Note that full information on the approval of the study protocol must also be provided in the manuscript.
